# Effects of Mode Mixity and Loading Rate on Fracture Behavior of Cracked Thin-Walled 304L Stainless Steel Sheets with Large Non-Linear Plastic Deformation

**DOI:** 10.3390/ma16247690

**Published:** 2023-12-18

**Authors:** Jamal Bidadi, Hamed Saeidi Googarchin, Alireza Akhavan-Safar, Lucas F. M. da Silva

**Affiliations:** 1 Automotive Fluid and Structures Analysis Research Laboratory, School of Automotive Engineering, Iran University of Science and Technology (IUST), Tehran 13114-16846, Iran; jamal.bidadi@gmail.com; 2 Institute of Science and Innovation in Mechanical and Industrial Engineering (INEGI), 4200-465 Porto, Portugal; aakhavan-safar@inegi.up.pt; 3 Department of Mechanical Engineering, Faculty of Engineering, University of Porto, 4200-465 Porto, Portugal

**Keywords:** mixed-mode ductile fracture, steel 304L, loading rate effect, mode mixity

## Abstract

This study investigates the mixed-mode I/II fracture behavior of O-notched diagonally loaded square plate (DLSP) samples containing an edge crack within the O-notch. This investigation aims to explore the combined effects of loading rate and mode mixity on the fracture properties of steel 304L, utilizing DLSP samples. The DLSP samples, made from strain-hardening steel 304L, were tested at three different loading rates: 1, 50, and 400 mm/min, covering five mode mixities from pure mode I to pure mode II. Additionally, tensile tests were performed on dumbbell-shaped specimens at the same loading rates to examine their influence on the material’s mechanical properties. The findings revealed that stress and strain diagrams derived from the dumbbell-shaped samples were largely independent of the tested loading rates (i.e., 1–400 mm/min). Furthermore, experimental results from DLSP samples showed no significant impact of the loading rates on the maximum load values, but did indicate an increase in the ultimate displacement. In contrast to the loading rate, mode mixity exhibited a notable effect on the fracture behavior of DLSP samples. Ultimately, it was observed that the loading rate had an insignificant effect on the fracture path or trajectory of the tested DLSP samples.

## 1. Introduction

In recent years, numerous studies have investigated the failure behavior of engineering components, considering the presence of notches and cracks [1,2,3,4,5,6,7]. While notches are sometimes intentionally designed in engineering parts [2,4,6,8,9,10,11,12,13,14,15,16,17], cracks are invariably considered undesirable. Cracks typically form during the manufacturing process or the operational life of a component, diminishing the load-bearing capacity of structures and consequently shortening their lifespan. Crack faces in components can experience loading in three distinct modes: pure mode I, involving tensile loading; pure mode II, characterized by shear loading; and pure mode III, associated with tear loading [18]. Additionally, they can encounter complex combinations of mixed-mode I/II, I/III, and II/III loading conditions [2]. Brittle or unstable fractures, which result from rapid crack growth, typically occur in brittle and quasi-brittle materials. Conversely, ductile or stable fractures, arising from slow crack growth, usually manifest in ductile materials that exhibit significant plastic deformation near the crack tip. As brittle fracture poses a greater risk than ductile fracture, substantial research efforts have focused on understanding brittle fracture in engineering materials and structures in recent years. Among engineering materials, thin-walled ductile sheets made of steel, aluminum, and other metallic materials are consistently utilized in the construction of various structures, notably in vehicles within the automotive and aerospace industries [13,16,19]. Given the extensive application of thin-walled metallic sheets, it is imperative to research and study the ductile fracture behavior of thin-walled metallic materials utilized in different sectors. Sheet metal forming is a crucial process in car body manufacturing, playing a central role in shaping and fabricating various components of a vehicle’s exterior [20,21,22,23,24,25,26,27,28]. This process involves transforming flat sheets of metal, typically steel or aluminum, into three-dimensional parts, which constitute the framework of a car body, by various techniques, such as stamping, deep drawing, and hydroforming. The primary objective of sheet metal forming in car manufacturing is to create components with precise dimensions and a high structural integrity. In sheet metal forming processes, the phenomenon of fracture, also known as sheet metal fracture or tearing, is a critical concern that can adversely affect the quality and integrity of formed components [25]. Fracture occurs when the metal sheet undergoes excessive deformation, resulting in the development of cracks or defects. Therefore, one key aspect of the fracture phenomenon in sheet metal forming is excessive deformation. Fracture often occurs when the sheet metal undergoes deformation beyond its material limits, leading to localized thinning and stretching. Additionally, the type of external applied loads or mode mixity can affect the fracture phenomenon in the sheet metal process, which can be divided into pure tensile, pure shear or a combination of tensile and shear loading conditions [29,30,31,32]. For example, shear fracture occurs along shear planes and is common in materials with low ductility, while the necking phenomenon, especially in regions with a high deformation, can make the material more susceptible to fracture. In addition to the mentioned factor, the speed of loading or loading rate is another important parameter that can affect the sheet metal forming process [22,23,25]. Higher loading rates, such as those encountered in rapid-forming processes, can increase the likelihood of fracture. In this regard, knowledge about the material properties for moderate loading rates from 1–500 mm/min, which is 0.016–8.3 mm/s [21], is therefore necessary for standard sheet metal forming processes. Therefore, it is necessary to enhance the specifications of the formability of materials, particularly considering the influence of the loading rate and mode mixity.

Extensive research has been conducted on the effect of loading rates on the tensile properties of thin-walled metallic sheets. Green et al. [33] investigated the impact of loading rates on the tensile stress, flow stress, and elongation of SAE1008 steel sheet metal forming. They demonstrated the high sensitivity of these parameters to loading rates. Taduka et al. [34] examined the formability of a thin sheet of Mg–8.5Li–1Zn through uniaxial tensile tests and reported a significant increase in elongation at failure, even at lower loading rates. In another study, Srinivas and Kamat [35] explored the effect of loading rates on the tensile properties and fracture toughness of mild steel. They observed that the tensile yield strength remained independent within small ranges of loading rates. However, beyond these rates, the tensile yield strength increased with the loading rate. Additionally, they found an increase in the mode I fracture toughness with an increase in the loading rate. Other researchers have also studied the influence of mode mixity on the ductile fracture behavior of thin-walled steel and aluminum sheets. Torabi et al. [36] conducted a study that investigated the effect of mode mixity on the ductile fracture behavior of cracked aluminum and steel sheets. Their research focused on materials exhibiting considerable plastic deformation and highlighted the significant influence of mode mixity on the fracture resistance of these sheets.

A review of the literature indicates a scarcity of investigations regarding the simultaneous effects of loading rates and mode mixity on the tensile properties and ductile mixed-mode fracture behavior of thin-walled steel sheets with considerable plastic deformation. The primary objective of this study is to experimentally investigate the effect of loading rates on the tensile properties and mixed-mode fracture behavior of thin-walled steel sheets. Specifically, the focus is on stainless steel 304L, known for exhibiting significant non-linear plastic deformations. This investigation considers mode mixities ranging from pure mode I to pure mode II crack deformations. In this context, dumbbell-shaped samples and O-notched diagonally loaded square plate (DLSP) samples containing an edge crack were employed for performing tensile and mixed-mode fracture tests under different loading rates, specifically 1, 50, and 500 mm/min. The results reveal that the tensile and fracture properties of the tested stainless steel 304L with large non-linear plastic deformations remain independent of loading rates (1–400 mm/min). However, mode mixity significantly influences fracture behavior. In other words, maximum loads remain consistent and ultimate displacements increase slightly when changing the loading mode from pure mode I to pure mode II. It should be noted that the findings of this research align with the outcomes presented in [36]. Torabi et al. [36] investigated the mixed-mode I/II fracture behavior of aluminum thin-walled sheets with a small plastic deformation, while the current research delves into high plastic deformation and loading rate changes in steel sheets.

## 2. Experimental Study

### 2.1. Material and Specimen

**Figure 1:**
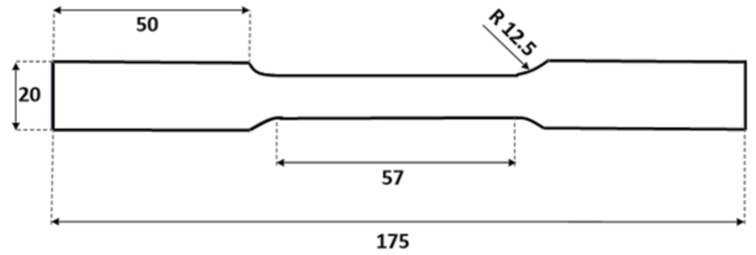
Schematic of the dumbbell-shaped specimen with 1 mm thickness (dimensions in mm).

**Figure 2:**
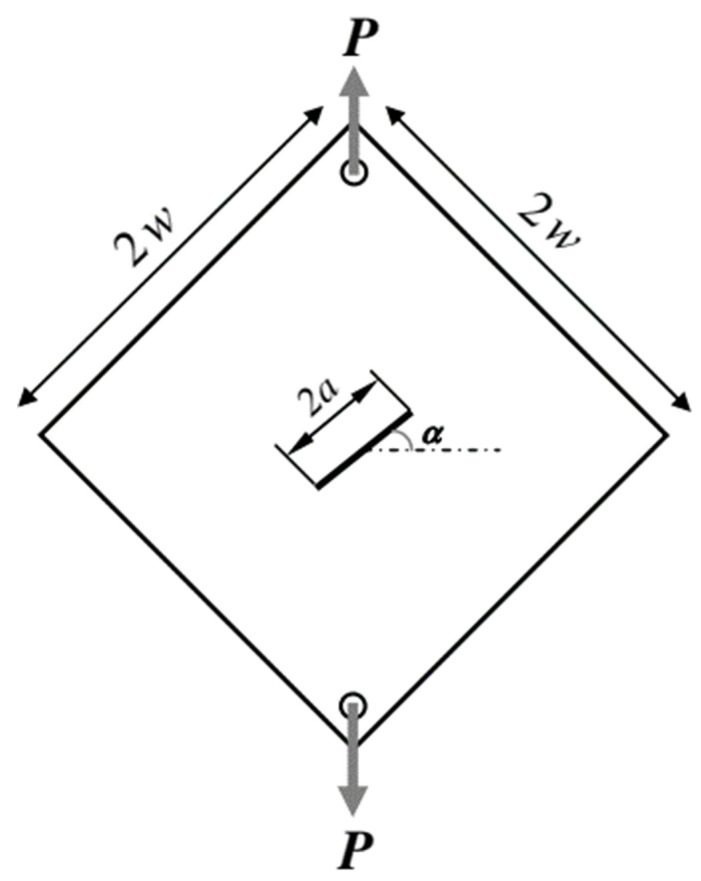
Schematic of the O-notched diagonally loaded square plate (DLSP) specimen with 1 mm thickness.

Figure 1 shows the tensile test samples used, according to the ASTM E8 [36] standard. For fracture testing, DLSP (O-notched diagonally loaded square plate) samples were utilized. The DLSP sample was first introduced by Torabi et al. [36] to analyze the mixed-mode ductile fracture behavior of thin-walled metallic materials. In this study, we adopted this specimen to investigate the loading rate dependency of mixed-mode ductile fracture behavior in thin-walled steel 304L sheets. As shown in Figure 2, the DLSP sample resembles a square, thin sheet of steel with a length of 2*W*. The desired square-shaped components along with their central grooves were cut using a two-dimensional numerical control computer in the form of a laser with oxygen, applied on a 1 mm thick plate. Subsequently, a wire cutter equipped with a 0.25 mm diameter wire was used to create symmetrical pre-cracks with a length of *a*. Figure 2 displays the geometry of the DLSP sample with pre-cracks, depicting its geometric parameters and associated boundary conditions. The values of the geometric parameters, including 2w, 2*a*, the specimen’s thickness, and the hole’s diameter, were 100, 60, 1, and 8 mm, respectively. Additionally, the angle of rotation of the crack in relation to the loading direction, denoted as alpha (α), assumed values of 0° for pure mode I, 15°, 30°, and 50° for mixed-mode I/II, and 67° for pure mode II degrees.

The crack tip parameters, including the dimensionless mode I (${Y_{I}}^{*}$) and mode II (${Y_{II}}^{*}$) stress intensity factors (shown below) and mode mixity for the DLSP sample, are provided in Table 1 from Ref. [36] and can be described as follows:

(1) $$\left\{\begin{array}{c} {Y_{I}}^{*}=\frac{K_{I}}{\frac{P}{Wt}\;\sqrt{\frac{\pi a}{2}}}\;\;\\ {Y_{II}}^{*}=\frac{K_{II}}{\frac{P}{Wt}\;\sqrt{\frac{\pi a}{2}}} \end{array}\right.\;\;\;\;\;\;{Y_{I}}^{*},\;{Y_{II}}^{*}\;=\;Function\;(a/W,\;\alpha )$$

(2) $$M\;=\;\frac{2}{\pi }\tan^{-1}\left(\frac{{Y_{I}}^{*}}{{Y_{II}}^{*}}\right)$$

where *P* is the applied load, *W* and *t* are the specimen width and thickness, and $K_{I}$ and $K_{II}$ are the stress intensity factors.

### 2.2. Fracture Test

As mentioned in the previous section, the loading mode of the DLSP sample can be altered by adjusting the alpha (α) angle. When alpha is set to zero, a pure mode I loading is achieved, whereas an alpha angle of 67° corresponds to a pure mode II loading. As mentioned before, all angles between 0° and 67° represent mixed-mode I/II loading conditions at the crack tip. Notably, the angle of 67 degrees, denoting the conditions for pure mode II, was derived from Torabi et al. [36]. Their extensive finite element simulations analyzed the DLSP specimen and its mode mixity across various alpha (α) angles. Fracture tests were conducted by subjecting cracked DLSP specimens to uniaxial uniform tension. The loading was displacement-controlled, and three loading rates (1, 50, and 400 mm/min) were selected for each of the five loading modes. Thus, a total of 45 tests were performed on the DLSP samples, covering fifteen different cases, with three repetitions for each test. Additionally, nine tests were conducted on dumbbell-shaped samples, with three repetitions at each loading rate (1, 50, and 400 mm/min). The outcome of each fracture test includes a load-displacement curve along with experimental observations on the specimen’s failure.

## 3. Results and Discussion

**Figure 3:**
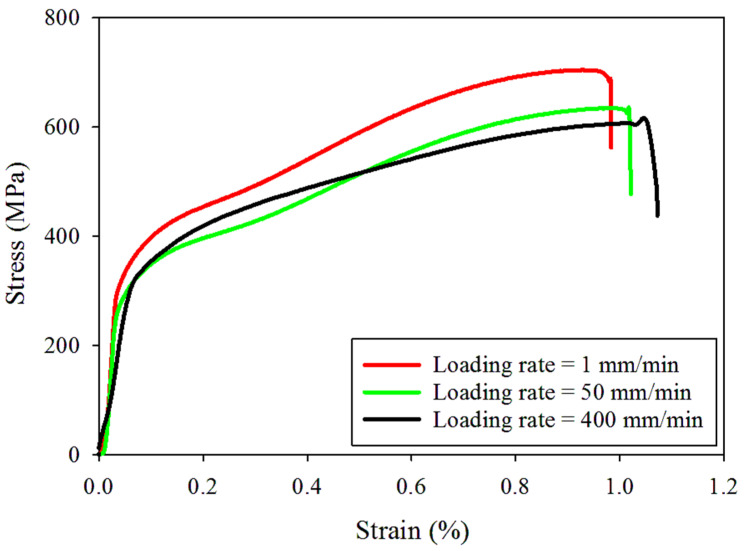
Stress–strain curves of the tested dumbbell-shaped samples under different loading rates.

**Figure 4:**
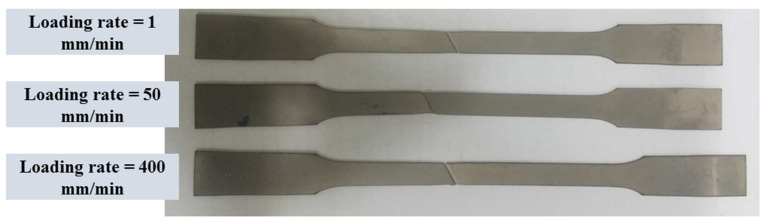
Fractured dumbbell-shaped samples under different loading rates.

The mechanical tests were conducted by employing a Santam (IRAN) universal testing machine with a loading capacity of 15 tons. Tensile and fracture loads were consistently applied at a controlled displacement rate, facilitating both tensile and fracture tests on dumbbell-shaped and cracked DLSP samples. The force-displacement data were precisely recorded using a digital data logging system. The stress–strain relationships were derived from uniaxial tension tests performed on dumbbell-shaped samples at constant crosshead displacements of 1, 50, and 400 mm/min. This section aimed to explore the influence of the loading rate on the tensile behavior of thin-walled stainless steel 304L sheets. In this regard, the engineering stress and strain curves and also the fractured dumbbell-shaped samples after tests are presented in Figure 3 and Figure 4. As shown in Figure 4, the changes in the stress–strain values concerning the loading rate are minimal, suggesting only a slight shift in both stress and strain. For example, the ultimate strength ($\sigma_{u}$) values decrease from 704 MPa to 606 MPa as the loading rate increases from 1 to 400, while the ultimate strain ($\varepsilon_{u}$) values increase from 0.98% to 1.12% with the rising loading rate. The observed pattern in the variation of ultimate strain ($\varepsilon_{u}$) values with the loading rate in the tested steel 304L sample is in accordance with the findings from a similar sample outlined in reference [37]. Additionally, as the loading rate goes from 1 to 50 mm/min, the yield strength ($\sigma_{y}$) decreases; however, from 50 to 400 mm/min, it shows an increase. Nonetheless, these changes remain insignificant. Additionally, it is notable that the slope of the stress–strain diagram in the linear region, indicating the material’s modulus of elasticity (*E*), remains relatively constant despite the increase in the loading rate. This trend suggests that the material’s softening does not change as the loading rate rises from 1–400 mm/min. Precise values for yield strength ($\sigma_{y}$), ultimate strength ($\sigma_{u}$), and ultimate strain ($\varepsilon_{u}$) parameters are detailed in Table 2.

**Figure 5:**
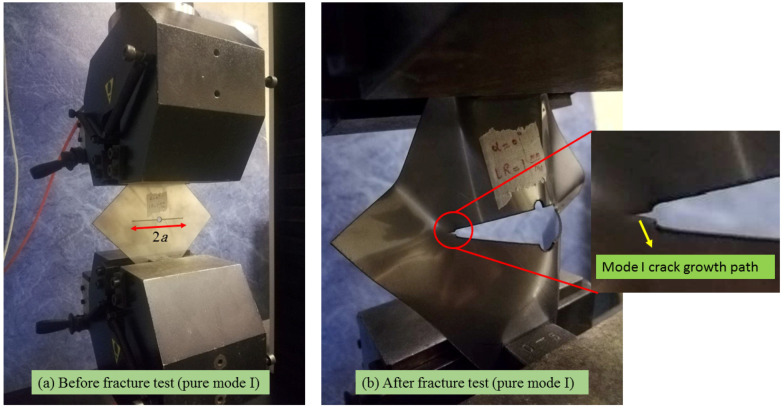
Cracked DLSP sample under pure mode I loading before and after a fracture test. (**a**) The DLSP sample before mode I fracture test, (**b**) The DLSP sample after mode I fracture test.

**Figure 6:**
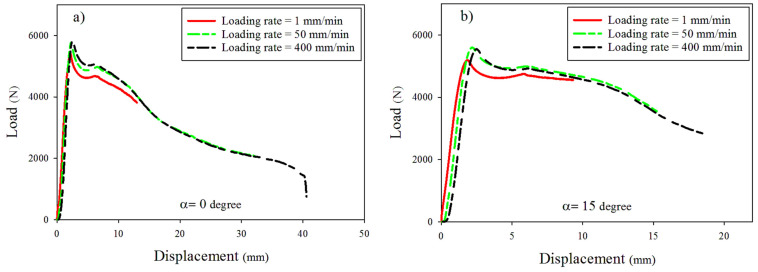

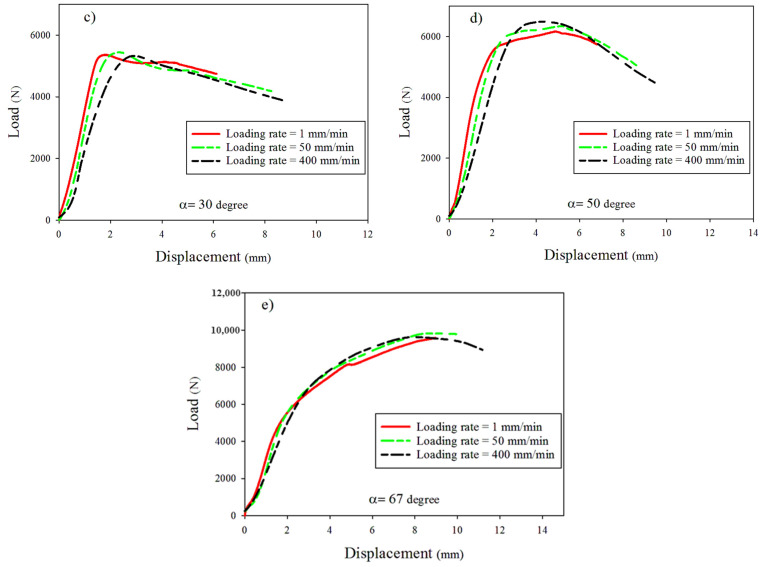
Typical load-displacement curves of the tested DLSP samples under different loading rates. (**a**) Pure mode I (M = 1), (**b**) Mixed-mode (M = 0.8), (**c**) Mixed-mode (M = 0.57), (**d**) Mixed-mode (M = 0.24), and (**e**) Pure mode II (M = 0).

A view of the DLSP sample in the test setup is illustrated in Figure 5. Additionally, Figure 6 indicates the results of force-displacement at different mode mixities including pure mode I (Figure 6a), mixed-mode I/II (Figure 6b–d), and pure mode II (Figure 6e) and loading rates (1, 50, 400 mm/min). According to Figure 5 and Figure 6, it was observed that the cracked DLSP specimen exhibited ductile fracture behavior characterized by substantial non-linear plastic deformation. In other words, during the early stages, the load increases linearly with the displacement and eventually shifts into a non-linear progression until it reaches its maximum value. After reaching the maximum load value, the samples continue deforming until reaching ultimate rupture.

**Figure 7:**
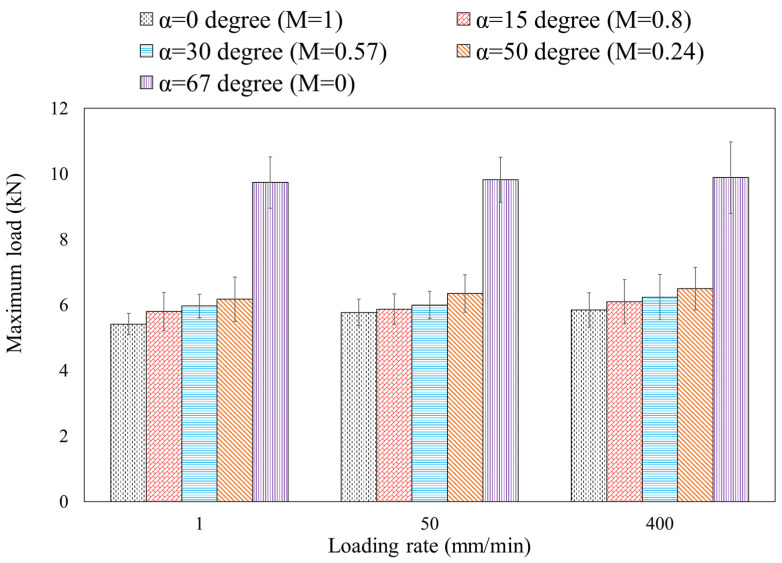
Variations of the maximum load for DLSP samples under different loading rates.

**Figure 8:**
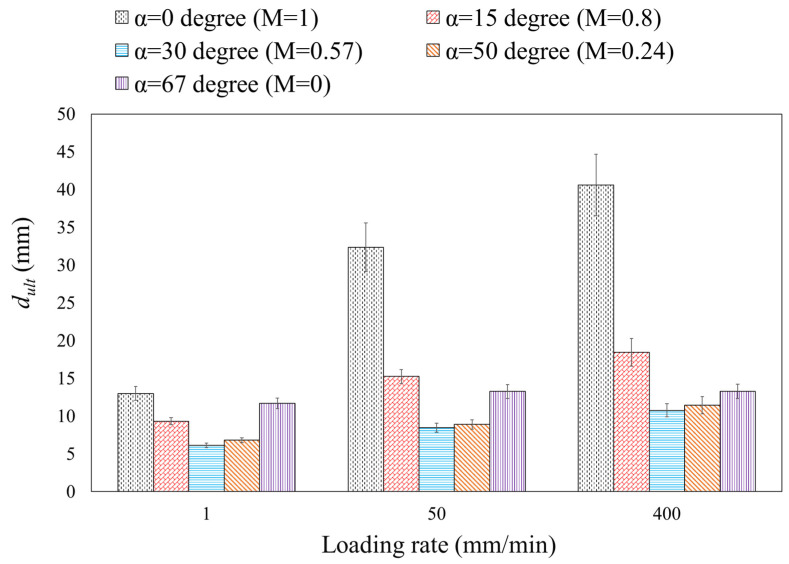
Variations of the ultimate displacement (*d_ult_*) for DLSP samples under different loading rates.

**Figure 9:**
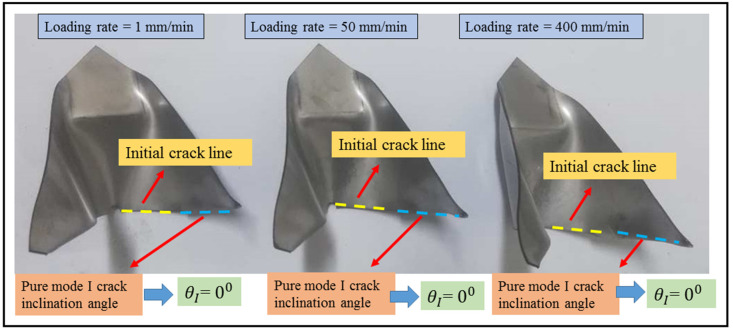
Pure mode I crack initiation angle for DLSP samples under different loading rates.

Additionally, there is a slight increase in maximum load values depicted in Figure 7 with increasing loading rates. For example, as the loading rate increased from 1 to 400 mm/min, the maximum load values for alpha (α) angles of 0, 15, 30, 50, and 67 increased by approximately 7.9%, 5.3%, 4.5%, 5.25%, and 2%, respectively. These findings suggest that the mixed-mode failure load in steel 304L DLSP samples remains largely independent of the loading rate within the tested range of 1–400 mm/min in this study. However, the ultimate displacement ($d_{ult}$) in DLSP samples is notably influenced by the loading rate, exhibiting an increase with the ascending loading rate, so that this increase is small for bigger mode mixities from M = 0.24 ($\alpha =50^{{}^{\circ}}$) to M = 0 ($\alpha =67^{{}^{\circ}}$). The variations in the maximum load ($F_{c}$) and the ultimate displacement ($d_{ult}$) of DLSP samples are detailed in Figure 8 and Figure 9.

**Figure 10:**
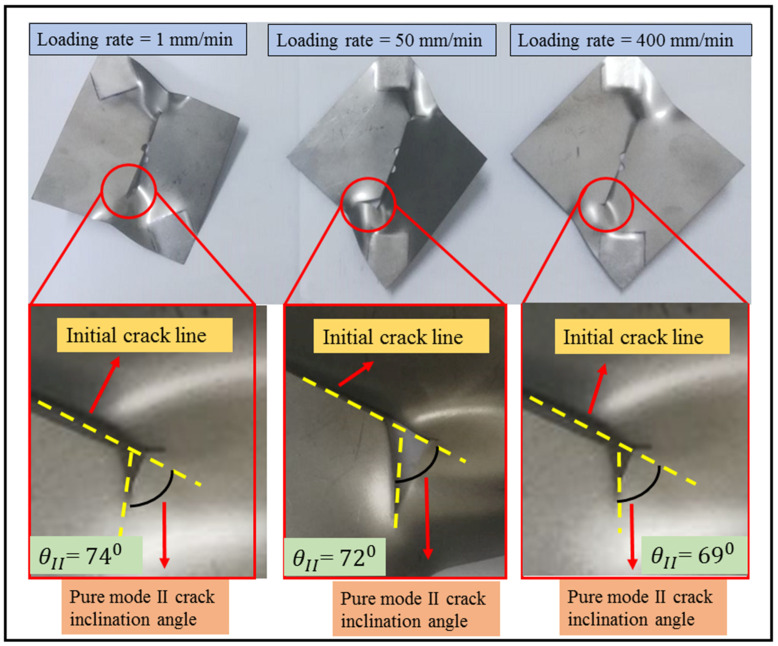
Pure mode II crack initiation angle for DLSP samples under different loading rates.

The bar graphs in Figure 7 clearly illustrate the independence of the maximum load of the DLSP sample from loading rates. As depicted in Figure 7, unlike the loading rate, the mode mixity (M) significantly influences the fracture behavior of DLSP samples. The maximum failure load increases as the mode mixity shifts from pure mode I (M = 1) to pure mode II (M = 0). For instance, the maximum load in pure mode II is 1.72 times greater than that in pure mode I. According to Figure 8, the relationship between ultimate displacement, loading rate, and mode mixity is evident. The increase in ultimate displacement with varying loading rates (1–400 mm/min) and for different mode mixities—M = 1, M = 0.8, M = 0.57, M = 0.24, and M = 0—is 207%, 89%, 102%, 93%, and 17%, respectively. Moreover, it is noticeable that for each specific loading rate, the ultimate displacement decreases from M = 1 to M = 0.57 and then increases again from M = 0.24 to M = 0. Overall, the ratio of the ultimate displacement in pure mode II to that in pure mode I remains consistent for the 1 mm/min loading rate. However, it decreases by about 62% and 70% for loading rates of 50 and 400 mm/min, respectively. Another noteworthy discovery is the negligible effect of the loading rate on the crack initiation angle of DLSP samples across various loading modes (M = 1, M = 0.8, M = 0.57, M = 0.24, and M = 0). The fracture path or trajectory in pure modes I and II under different loading rates is presented in Figure 9 and Figure 10. Upon observing Figure 9 and Figure 10, it becomes apparent that the loading rate (ranging from 1 to 400 mm/min) has an insignificant impact on the crack initiation angles for modes I and II. Specifically, the mode I and mode II crack initiation angles ($\theta_{I}$ and $\theta_{II}$) remain consistent across different loading rates, maintaining values of approximately 74, 72, and 69 degrees for pure mode II at loading rates of 1, 50, and 400 mm/min, respectively. It should be noted that the values of the crack initiation angles in Figure 9 and Figure 10 are obtained by measuring the angle between the pre-crack line and the direction of the crack growth. The angles measured here align consistently with those documented in previous references, such as ref. [36]. 

## 4. Conclusions

This study examined the tensile behavior and fracture response of steel 304L sheets under various loading rates and at different mode mixities. 304L stainless steel demonstrates substantial plastic deformation capabilities compared to other metallic materials. For tensile and fracture analysis of this material, dumbbell-shaped and pre-cracked DLSP samples were prepared and subjected to tensile tests at loading rates of 1, 50, and 400 mm/min. The findings indicated that the stress and strain diagrams derived from the dumbbell-shaped samples were nearly independent of the loading rates tested in this study (i.e., 1 to 400 mm/min). Moreover, the results of fracture tests on the pre-cracked DLSP samples revealed that the loading rates investigated did not significantly impact the maximum load values in the DLSP samples. The primary discernible effect of the loading rate manifested in the ultimate displacement of the DLSP samples, notably increasing with higher rates (especially in dominant mode one loading). In contrast to the loading rate, a substantial influence of the mode mixity (*M*) on the maximum load and ultimate displacement values was observed. Transitioning from pure mode I to pure mode II resulted in increased maximum load values and decreased ultimate displacement values, respectively. It was noted that elevating the loading rate led to an increase in displacement at failure, particularly in fracture tests, as observed in similar studies on analogous alloys subjected to varying strain rates. Another noteworthy discovery is the negligible effect of the loading rate on the crack initiation angle of DLSP samples across various loading modes.

## Figures and Tables

**Table 1:** Dimensionless values of mode I and mode II stress intensity factors for the DLSP specimen.

*M*	Loading Mode	α (*Degree*)	Y_I_*	Y_II_*
1	Mode I	0	1.21	0
0.8	Mixed-mode I/II	15	1.10	0.35
0.57	Mixed-mode I/II	30	0.78	0.61
0.24	Mixed-mode I/II	50	0.31	0.75
0	Mode II	67	0	0.58

**Table 2:** Mechanical properties of tested 304L steel.

Property	Loading Rate (mm/min)		
	1	50	400
$\mathrm{Tensile}\;\mathrm{yield}\;\mathrm{strength},\;\sigma_{y}$ (MPa)	280 ± 4	259 ± 3.5	305 ± 3
$\mathrm{Tensile}\;\mathrm{ultimate}\;\mathrm{strength},\;\sigma_{u}$ (MPa)	704 ± 3	635 ± 5	606 ± 4.3
$\mathrm{Rapture}\;\mathrm{strain},\;\varepsilon_{u}$ (%)	98 ± 6	105 ± 9	108 ± 8

## Data Availability

Data is contained within the article.
